# Alkaline-Metal Cations Affect Pt Deactivation for the Electrooxidation of Small Organic Molecules by Affecting the Formation of Inactive Pt Oxide

**DOI:** 10.1021/jacs.4c09590

**Published:** 2024-09-26

**Authors:** Victor Y. Yukuhiro, Rafael A. Vicente, Pablo S. Fernández, Angel Cuesta

**Affiliations:** † Chemistry Institute, Universidade Estadual de Campinas (UNICAMP), 13083-970 Campinas, São Paulo, Brazil; ‡ Center for Innovation on New Energies (CINE), Universidade Estadual de Campinas, 13083-841 Campinas, São Paulo, Brazil; § Advanced Centre for Energy and Sustainability (ACES), School of Natural and Computing Sciences, University of Aberdeen, AB24 3UE Aberdeen, Scotland, U.K.; ∥ Centre for Energy Transition, University of Aberdeen, King’s College, AB24 3FX Aberdeen, Scotland, U.K.

## Abstract

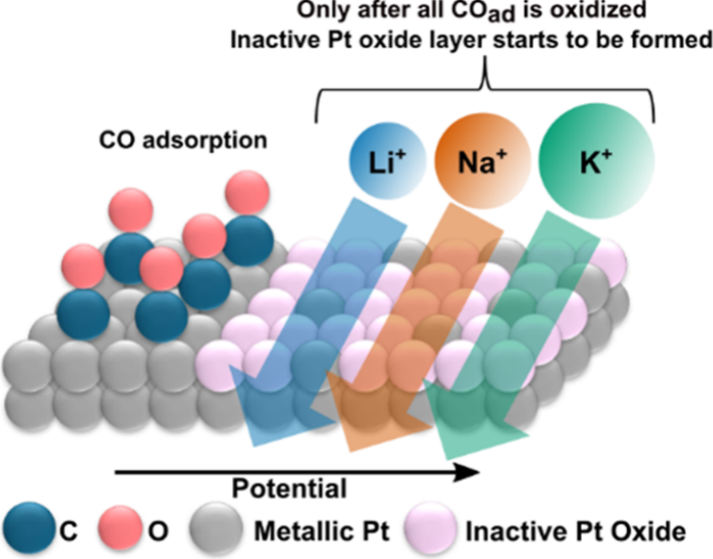

The activity of Pt for the electro-oxidation of several organic molecules changes with the cation of the electrolyte. It has been proposed that the underlying reason behind that effect is the so-called noncovalent interactions between the hydrated cations and adsorbed OH (OH_ad_). However, there is a lack of spectroscopic evidence for this phenomenon, resulting in an incomplete understanding at the microscopic level of these electrochemical processes. Herein, we explore the electro-oxidation of glycerol (EOG) on platinum (Pt) in LiOH, NaOH and KOH using *in situ* surface-enhanced infrared absorption spectroscopy in the attenuated total reflectance mode (ATR-SEIRAS) and *in situ* X-ray absorption spectroscopy (XAS). Our results show that the electrolyte cation influences the rate and potential at which adsorbed CO (CO_ad_), a catalytic poison, is formed and oxidized. We attribute this to the cation-dependent stability of oxygenated species on the metallic Pt surface and the different intensities of the electric field at the electrode/electrolyte interface. We also demonstrate that the formation of an inactive Pt oxide layer is indirectly also cation-dependent: the formation of this layer is triggered by the cation-dependent oxidative removal of reaction intermediates (for instance, CO). This phenomenon explains the well-known cation-induced differences in the voltammetric profiles, of not just glycerol, but generally of alcohols and polyols.

## Introduction

The electro-oxidation of glycerol (EOG) using noble metals such as Pt, Au, and Pd has been an area of intense research in recent decades.^1,2^ Glycerol (GlOH) is an abundant resource that finds use in fuel cells and electrolyzers, to generate electrical energy or produce high-purity hydrogen and high-value-added products,^3−7^ respectively.

The EOG on Pt is a complex process, which is influenced by various factors such as surface orientation,^8−11^ surface modification,^9,11−13^ and pH.^14^ In addition, the effect of cations in the electrolyte has been studied in the electro-oxidation of GlOH^15,16^ and other small organic molecules, like methanol,^17^ formate^18^ and ethylene glycol,^19^ on Pt. In all cases, the current density was observed to increase when going down the alkaline metals group. This phenomenon has been rationalized through the noncovalent interactions between the hydrated alkaline-metal cation and adsorbed OH (OH_ad_) on the Pt surface, which results in OH_ad_–M^+^(H_2_O)_*x*_ clusters that are “quasi-specifically adsorbed” on Pt, blocking surface sites and, therefore, hindering the reaction.^20^ Strmcnik et al.^17^ first proposed this hypothesis while studying the hydrogen oxidation and evolution (HOR and HER, respectively) as well as the methanol oxidation reactions (MOR) on Pt(111). They proposed that the higher hydration energy of smaller cations such as Li^+^ leads to the formation of a higher quantity of more stable clusters, blocking access of the reactive species to the active sites and, ultimately, acting as a catalyst poison. However, no spectroscopic evidence was provided or has been provided since to support this hypothesis.

Melle et al.^16^ investigated the EOG in alkaline media using aqueous electrolytes containing XOH, where X = Li^+^, Na^+^ and K^+^. To do so, they combined electrochemical experiments with high-performance liquid chromatography (HPLC). They observed activity trends (Li^+^ < Na^+^ < K^+^) consistent with the results reported by Angelucci et al.^15^ Additionally, they identified the products formed during the reaction as glycerate and lactate, in agreement with de Souza et al.^12^ and Lima et al.,^21^ who used polycrystalline Pt and Pt/C, respectively, in NaOH solution. Overall, these results suggest that the electrolyte cation has a greater impact on the electrochemical activity than on product selectivity.

The formation of adsorbed intermediates, among them carbon monoxide (CO_ad_), the most commonly detected poisoning reaction intermediate, is a critical aspect of the electro-oxidation of organic molecules on Pt.^22−24^ Therefore, investigating the impact of different cations on the nature and dynamics of the formation and oxidation of this adsorbed intermediate species is essential for gaining a better understanding of the mechanism and activity of the EOG.

In this study, we employed *in situ* surface-enhanced infrared absorption spectroscopy in the attenuated total reflectance mode (ATR-SEIRAS) to investigate at the molecular level the effect of electrolyte cations on the EOG on Pt in alkaline media. We mainly focused on following CO_ad_ formation and oxidation, since signals from nonadsorbed products from partial and complete oxidation of GlOH have poor signal intensity due to the short-range of the SEIRAS effect. Our results show that changing the electrolyte cation changes the potential for the formation and oxidation of CO_ad_. Besides, as we go down the alkaline metals group, the enhanced OH adsorption and the stabilization effect on OH_ad_ are less intense.

Finally, a well-known observation already commented on here is that platinum’s electrocatalytic activity for the oxidation of several alcohols and polyols follows the same trend, K^+^ > Na^+^ > Li^+^, as the EOG. We observed that the main cation-induced differences in the CVs occur in the region of the catalyst’s deactivation, which we propose is due to the formation of inactive Pt oxide species and which is affected by the effect of the cation on the formation and oxidative removal of CO_ad_. To shed some light on this phenomenon, we performed *in situ* X-ray absorption spectroscopy (XAS), giving evidence that this deactivation occurs indeed due to this phenomenon, and not due to the steric effect resulting from the different noncovalent interactions between the cations and OH_ad_, as commonly held.

## Experimental Section

For the ATR-SEIRAS experiments, the solutions were prepared by dissolving LiOH monohydrate (99.995% metal basis, Thermo Scientific), NaOH monohydrate (99.996% metal basis, Thermo Scientific) or KOH (99.98% trace metal basis, Thermo Scientific), in ultrapure water (18.2 MΩ cm, Elga Veolia) to yield 0.5 M solutions. Solutions containing glycerol (GlOH) were prepared by adding GlOH (≥99.5%, Sigma-Aldrich) to the desired concentration.

For the *in situ* XAS experiments, the solutions were prepared by dissolving LiOH (anhydrous, 99.9% trance metal basis, Sigma-Aldrich) or KOH (semiconductor grade pellets, 99.99% trace metal basis, Sigma-Aldrich) in ultrapure water (18.2 MΩ cm, Millipore) to yield 0.5 M solutions. GlOH (≥99.5%, Sigma-Aldrich) was added to the solution in the desired concentration.

All ATR-SEIRAS experiments were performed after deaeration of the electrolyte by purging N_2_ at room temperature. The working electrode (WE) was a Pt film deposited on the surface of a Si triangular prism beveled at 60° following a procedure reported elsewhere.^25^ The counter electrode (CE) was a flame-annealed Pt mesh and the reference electrode (RE) was a homemade reversible hydrogen electrode (RHE), to which all potentials in the text are referred unless otherwise stated. The electrochemical surface was calculated using the charge in the hydrogen adsorption–desorption region, taking 210 μC cm^–2^ as the charge corresponding to a flat polycrystalline Pt surface.^26^ Infrared spectra were recorded with a Nicolet iS50R FT-IR (Thermo Fisher Scientific) spectrometer equipped with a liquid N_2_-cooled mercury cadmium telluride (MCT) detector and a homemade ATR accessory. Once covered with the Pt film, the Si prism was attached to the spectroelectrochemical cell with an O-ring to seal the system. Electric contact with the WE was made by pressing a Pt circular wire onto the surface of the Pt film. All spectra are represented in absorbance units (a.u.) defined as , where *R*_background_ is the background spectrum (collected at 0.05 V_RHE_ before the addition of GlOH to the solution) and *R*_sample_ is the spectrum in the presence of GlOH at the sample potential, usually recorded in real-time during a cyclic voltammogram (CV).

In all ATR-SEIRAS experiments, the potential was controlled using a CHI600C potentiostat. To test the cleanliness of the system, we first recorded the CV of the Pt film in the absence of GlOH between 0.1 and 1.4 V_RHE_ at 100 mV s^–1^ (Figure S1). The WE was then polarized at 0.05 V_RHE_ and the background spectrum, consisting of 64 interferograms with a spectral resolution of 8 cm^–1^, was recorded. Then, GlOH was added to a final concentration of 0.1 M and we waited 120 s before starting the collection of the spectral series, which was obtained in real-time during a CV at 10 mV s^–1^ by accumulating 4 interferograms per spectrum with a spectral resolution of 8 cm^–1^, resulting in a time interval of ∼0.56 s, with each spectrum scanning over 5.6 mV. All the results shown here correspond to the first voltammetric cycle in the presence of GlOH.

The XAS experiments were performed in fluorescence mode at the nanoprobe beamline (CARNAÚBA) at Sirius (LNLS). For these experiments, the beam size is roughly 200 × 500 nm^2^. The in situ experiments were carried out using a SRS-EC301 potentiostat to control a purpose-built spectroelectrochemical cell collecting XANES spectra at target potentials.^27^ The WE was a glassy carbon disk (Ø = 1 mm) onto which we deposited 2.5 μL of the Pt ink.^28^ The XAS spectra were collected at the Pt L_III_ edge in energy steps of 0.5 eV. Sirius storage ring is operated at 100 mA. Most spectra shown here are an average of 2 or 3 subsequent ones, depending on the final signal-to-noise ratio. They are normalized using linear functions before (the initial 15 eV of the baseline) and after (the final 60 eV of the first extended X-ray absorption fine structure (EXAFS) waves) the edge. Each individual spectra took in average 3 min to be collected. All spectra were obtained using a fluorescence detector in reflection mode.

Probing the decrease of Pt’s electron density associated with the formation of the surface oxide layer with XAS is quite challenging for two reasons. First, the highly penetrating hard X-rays probe a good portion of the bulk of Pt in addition to the thin surface oxide layer. Second, KOH and LiOH are expected from the information contained in the corresponding CVs to form oxides only a few tenths of millivolts apart. Thus, in these regions, only a very small amount of oxide/oxygenated adsorbed species is present on the Pt surface (i.e., the spectrum is even more dominated by contributions from bulk Pt). In order to overcome these challenges, we used Pt nanoparticles with sizes around 3 nm^28^ to maximize the surface/volume ratio. In addition, all XAS measurements were performed at the Carnaúba nanoprobe beamline in Sirius. Using a nanometric beam, we can probe only some micrometric agglomerates of nanoparticles, increasing the sensitivity of the measurement. By doing this, we were able to detect *in situ* the onset of the oxygen adsorption as a function of the applied potential by tracking the intensity of the white line (WL) of Pt.

## Results

### Cyclic Voltammetry

**Figure 1:**
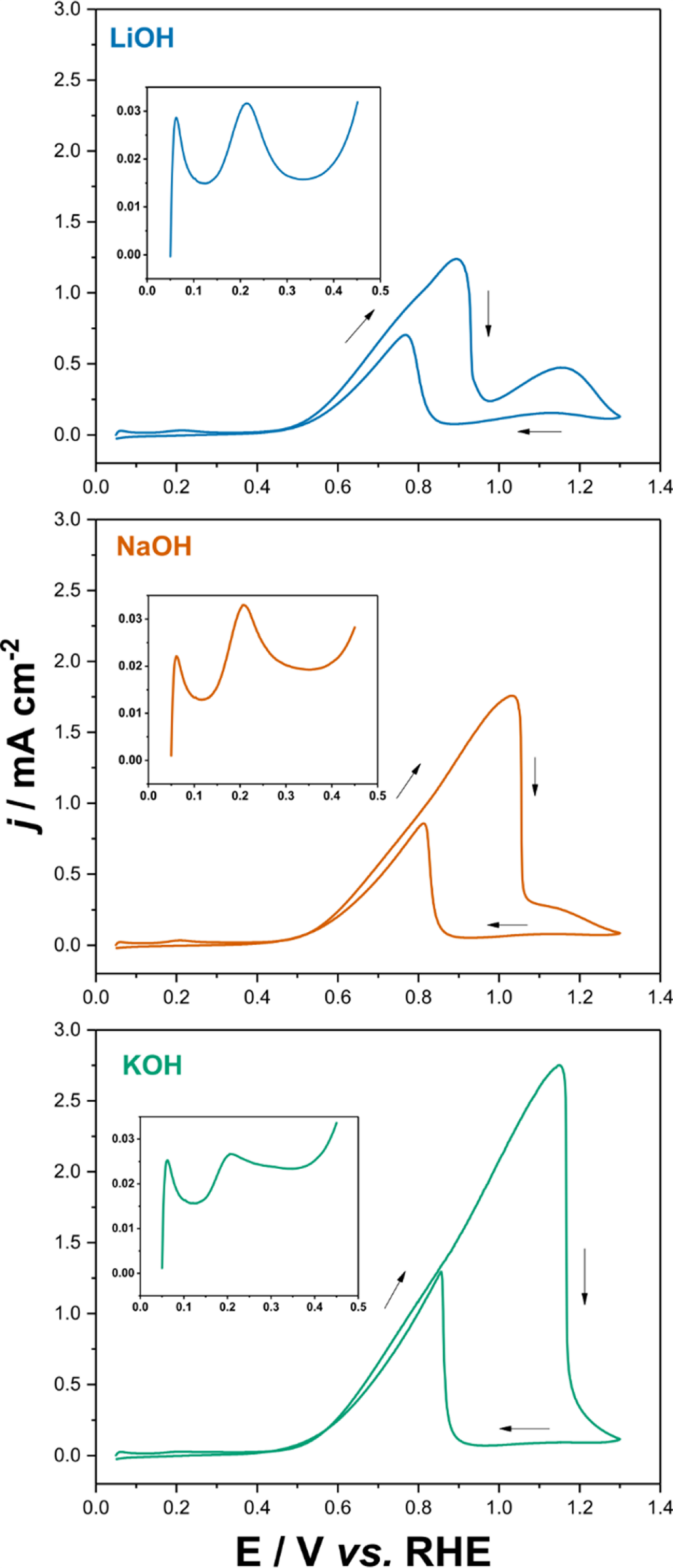
Typical cyclic voltammograms of Pt thin-film electrodes on silicon in 0.1 M GlOH + 0.5 M XOH, where X = Li (up), Na(mid), and K(bottom) at 10 mV s^–1^. Zooms into the region between 0.05–0.45 V_RHE_ are shown in the corresponding insets.

Typical CVs of the EOG on Pt thin-film electrodes in different aqueous electrolytes (XOH, where X = Li, Na, and K) are presented in Figure 1. The voltammetric profile is similar in all cases, although the main oxidation peak in the positive-going sweep shifts positively and its peak current density increases as we go down the alkaline metals group, Li^+^ < Na^+^ < K^+^. The anodic peak in the negative-going scan also shifts positively as we go down the group. All CVs are affected by the ohmic drop due to the resistance of the thin Pt film deposited on Si. However, this contribution is similar in all three electrolytes employed, as proven by the similar slope of the linearly increasing current positive of 0.5 V_RHE_ in all three CVs.

These results are consistent with the literature.^15,16^ As mentioned before, the increase in electrochemical activity as we descend from Li^+^ to K^+^ has been explained by the noncovalent interactions between the hydrated cations and OH_ad_ on the Pt surface, forming clusters that partially block the surface and limit the access of the reactant to the electrode surface.^15,20^ This should lead to a decrease in the current density with increasing hydration energy of the cation (hydration energy of K^+^ < Na^+^ < Li^+^), as indeed observed. However, as we will show below, our ATR-SEIRAS and XAS results reveal that the observed activity trend is not related to the steric effect resulting from the formation of such clusters.

Another feature common to all cations is shown in the insets in Figure 1, namely, a peak at 0.213, 0.208, and 0.206 V_RHE_ for LiOH, NaOH, and KOH, respectively. This peak has been attributed to the oxidation of GlOH to CO_ad_,^23,29,30^ a process that we will explore below in more detail based on our new ATR-SEIRAS results.

A final cation effect worth mentioning is the positive shift of the H_upd_ peaks as we go down the alkaline metals group (Figure S2), a trend already reported both with single-crystal and polycrystalline Pt electrodes.^31−33^ This is in agreement with the higher stabilization of OH_ad_ following the sequence Li^+^ > Na^+^ > K^+^, as these peaks are not just due to the adsorption/desorption of H_upd_, but rather to the replacement of H_upd_ by OH_ad_.^34,35^

### ATR-SEIRAS

**Figure 2:**
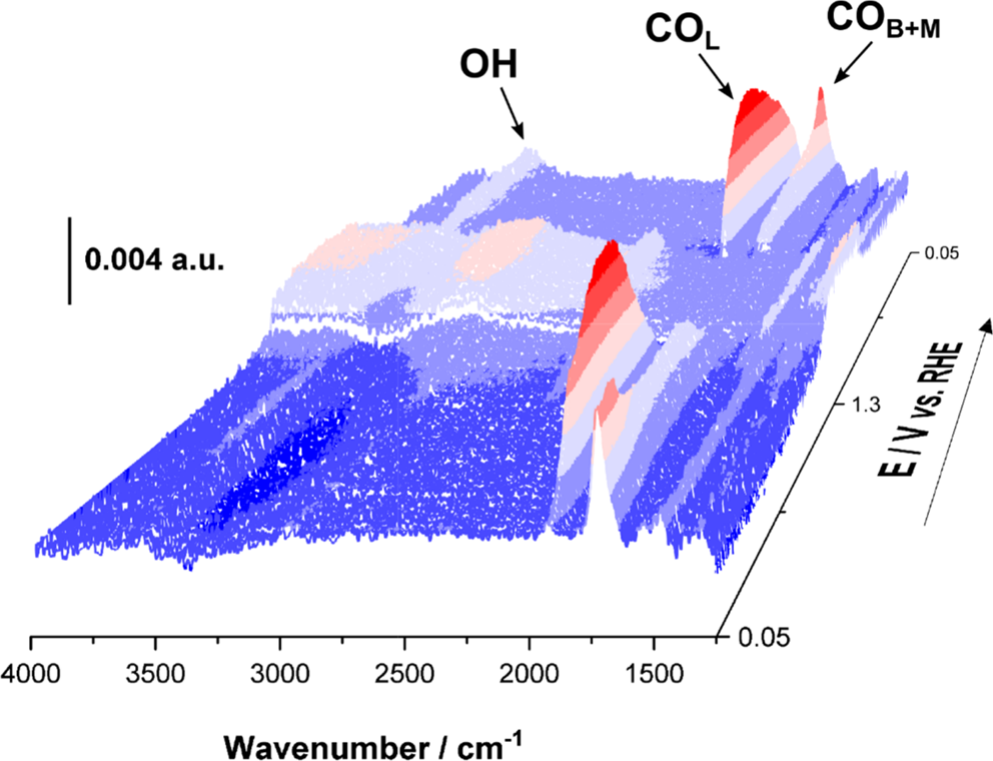
Typical time-resolved ATR-SEIRA spectral series obtained in 0.5 M KOH + 0.1 M GlOH during a cyclic voltammogram at 10 mV s^–1^ between 0.05 and 1.30 V_RHE_. Each spectrum is composed of 4 interferograms with a spectral resolution of 8 cm^–1^. The background is composed of 64 interferograms with a spectral resolution of 8 cm^–1^ and was acquired at 0.05 V_RHE_ in the absence of GlOH.

To obtain information at the molecular level of the interfacial processes associated with each of the voltammetric features in Figure 1, we recorded ATR-SEIRA spectral series synchronized with CVs between 0.05 and 1.30 V_RHE_ at 10 mV s^–1^ in 0.5 M XOH (X = Li, Na and K) + 0.1 M GlOH. Spectra in the series are the result of accumulating 4 interferograms with a time interval of ∼0.56 s between spectra, *i.e*., each spectrum spans a potential interval of 5.6 mV. For the sake of clarity, we only show in Figure 2 the series corresponding to the experiment in KOH. Series with LiOH and NaOH are shown in Figures S3 and S4, respectively.

The spectra in Figure 2 were collected during the first positive-and subsequent negative-going scans, and contain bands (i) in the region around 3500 cm^–1^ corresponding to the OH stretching of interfacial water, (ii) in the region between 2040 and 1700 cm^–1^, corresponding to CO_ad_, (There are different types of CO_ad_ coordination with the Pt surface, being: bridge-bonded and multiply bonded CO_ad_ (CO_B+M_), linearly bonded CO_ad_ (CO_L_) which are schematically represented in Figure S5.) (iii) around 1650 cm^–1^, corresponding to the bending mode of interfacial water and the C=O stretching modes of ketones and aldehydes, and (iv) in the region between 1600 and 1300 cm^–1^, which may contain vibrational modes due to the symmetric and asymmetric stretching modes of carboxylates (the latter only active if not adsorbed on the surface) as well as of CO_3_^2–^, the final product of the complete oxidation of glycerol. Additionally, a nonresonant contribution observed as an increase of the absorption baseline (more intense at higher frequencies) can be observed at potentials above 1.15 V_RHE_ due to the formation of a Pt oxide layer on the surface (please note that the presence of GlOH results in a cation-dependent positive shift of the potential at which Pt is oxidized, as will be shown below).

**Figure 3:**
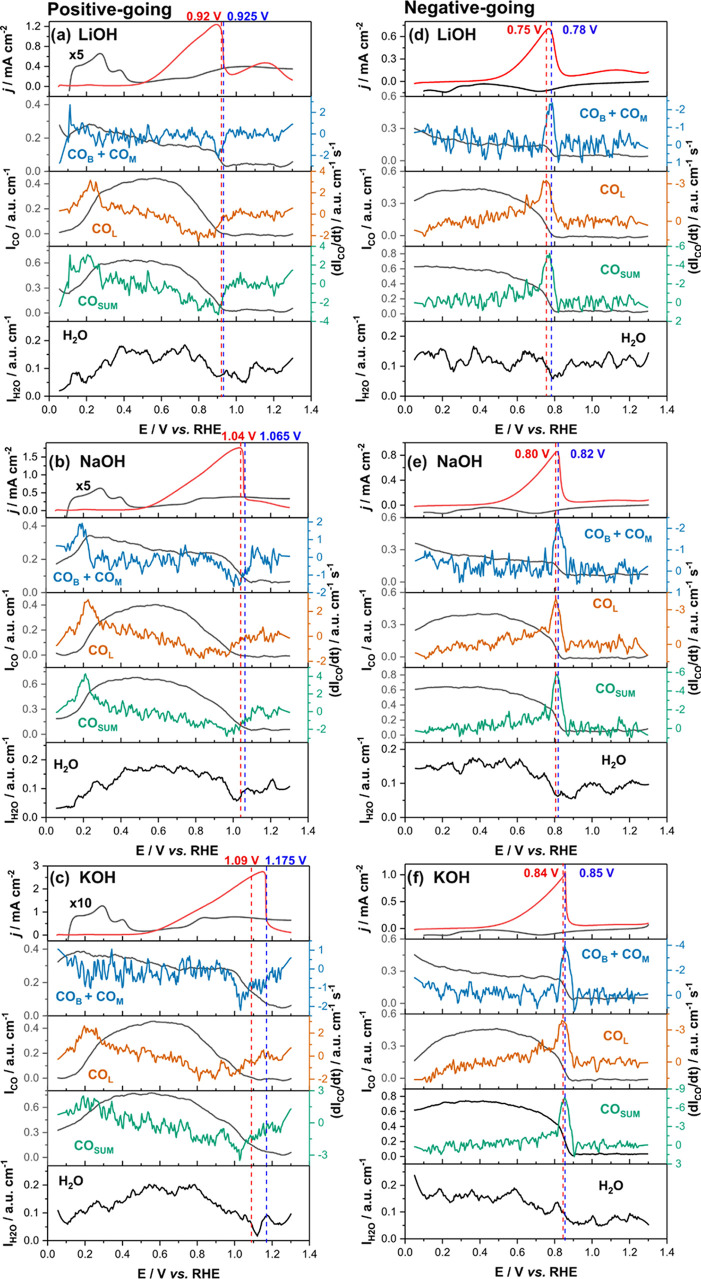
Spectroscopy-based analysis of the change in CO coverage in 0.5 M LiOH, NaOH, and KOH electrolytes during the positive- (a–c) and negative-going (d–f) scan of the cyclic voltammogram. Top panel: Voltammogram of Pt in the absence (black) and presence (red) of 0.1 M GlOH in 0.5 M XOH (X = Li, Na, and K) at 100 and 10 mV s^–1^, respectively. Other panels: Integrated intensity (black line) and corresponding derivatives (colored lines). For the positive-going scan, the dashed lines show the potential where all CO_L_ (red) and CO_B+M_ (blue) are oxidized. For the negative-going scan, the red and blue dashed lines are related to the peak of the formation rate of CO_L_ and CO_B+M_, respectively. See Figures S7–S9 in the Supporting Information for the unsmoothed integrated intensity data.

A more thorough description of the formation of CO_ad_ during GlOH oxidation can be achieved by a detailed comparison of the evolution of the integrated intensity of the CO_B+M_ and CO_L_ bands (which must be related to *θ*_CO_), as well as of the derivative of the integrated intensity (which must be related with the rate of formation of CO_ad_), during the CV (Figure 3). Because *θ*_CO_ is only proportional to the integrated intensity of the C–O stretching band(s) at low coverage due to dipole–dipole interactions at high coverage,^36^ it is helpful to also look at the potential dependence of the CO_L_ stretching frequency (as it increases with both potential and coverage^36^) and to the potential dependence of the intensity of the OH stretching band at ca. 3600 cm^–1^, as this is characteristic of interfacial water on CO-covered Pt.^37^

Figure 3(a,d),(b,e),(c,f) shows data for LiOH, NaOH, and KOH, respectively. The top panels in Figure 3(a–c) display the positive-going scans of the CV in the absence (black line) and presence (red line) of GlOH, whereas Figure 3(d–f) shows the corresponding negative-going scans. The remaining panels exhibit the potential dependence of the integrated intensity of the CO_ad_ bands (black lines) and the rate of formation/consumption (derivative of the intensity, colored lines) of, from top to bottom, CO_B+M_, CO_L_ and their sum (CO_SUM_). Finally, the bottom panels show the potential dependence of the integrated intensity of the OH stretching band of the CO_ad_-associated interfacial water. The intensity profile of this band closely resembles that of CO_SUM_, confirming that the latter rather accurately describes the evolution of the total CO coverage. The vertical red and blue dashed lines in Figure 3(a–c) mark the approximate potential at which CO_L_ and CO_B+M_, respectively, have been completely oxidized in the positive-going scan. Individual spectra around the potential at which CO_L_ and CO_B+M_ are fully oxidized, are shown in Figure S6. The vertical red and blue dashed lines in Figure 3(d–f) mark the peak potential in the derivative of the integrated intensity of the CO_L_ and CO_B+M_ bands, respectively, (*i.e*., the potential at which the rate of formation of CO_L_ and CO_B+M_ is maximum in the negative-going scan).

The intensity of the band corresponding to CO_L_ is negligible at 0.05 V_RHE_, and a clear CO_L_ band (1930–2030 cm^–1^, depending on potential and coverage) only emerges between 0.1 and 0.2 V_RHE_. This is not due to an incomplete reaction at 0.05 V_RHE_, as we do not observe any change in the spectral signature after prolonged polarization at this potential. On the contrary, the absence of a CO_L_ band is due to the relatively low CO coverage (θ_CO_) achieved by the oxidation of GlOH and the expectedly large negative charge density on the electrode surface (the potential of zero charge (pzc) of CO-covered polycrystalline Pt is 0.87 V_SHE_^38^) at this potential, as these two factors will both favor CO_B+M_ over CO_L_ on polycrystalline Pt.^24,39−41^ As the potential increases, so does the intensity of the CO_L_ band, which grows at the expense of the CO_B+M_ band and eventually becomes dominant.

Although all the systems show similar voltammetric and CO_ad_-intensity profiles, there are clear cation-induced differences in the positive-going scan (Figure 3(a–c)). For example, while the oxidation of CO_ad_ is complete in LiOH at 1.0 V_RHE_, 1.1 V_RHE_ is needed for a complete oxidation in NaOH and KOH. In all cases, though, when a potential positive enough for CO_ad_ to be oxidized is reached, CO_L_ disappears faster and more positive potentials are needed to completely oxidize CO_B+M_, which coincides with the drop of the current density in the CV. We attribute this current drop to the formation of an inactive Pt oxide layer. In the negative-going scan (Figures 3(d–f) and S8), the electrolyte cation also affects (i) the potential at which CO_ad_ starts to form again, (ii) the potential at which the rate of CO_ad_ formation peaks and (iii) the ratio between CO_B+M_ and CO_L_ at the negative potential limit.

The cation dependence of the potential at which the Pt surface deactivates by forming an inactive Pt oxide layer seems to follow a trend opposite to that expected from blank voltammograms. For instance, the onset of Pt oxidation is more negative in KOH than in LiOH (Figure S2). However, such comparison ignores the effect that the presence of the reactant, the reaction intermediates and the products may have on the process of formation of the platinum oxide layer. In this context, we used XAS to monitor changes in the electronic density of Pt during the EOG, aiming to track the formation of Pt oxides in the presence of this organic molecule.

### XAS

XAS absorption is a powerful tool to probe changes in the electronic density. The electrochemically driven oxidation of Pt is preceded by the adsorption of OH, which is then converted to a Pt oxide layer. In both of these steps, the bond between oxygen and Pt depletes the latter’s d orbitals, decreasing the electronic density in the Pt surface. As the d orbitals lose electron density, the white line (WL) intensity observed in an XAS spectrum increases, as there are more empty orbitals for a 2p-5d transition.^42,43^

**Figure 4:**
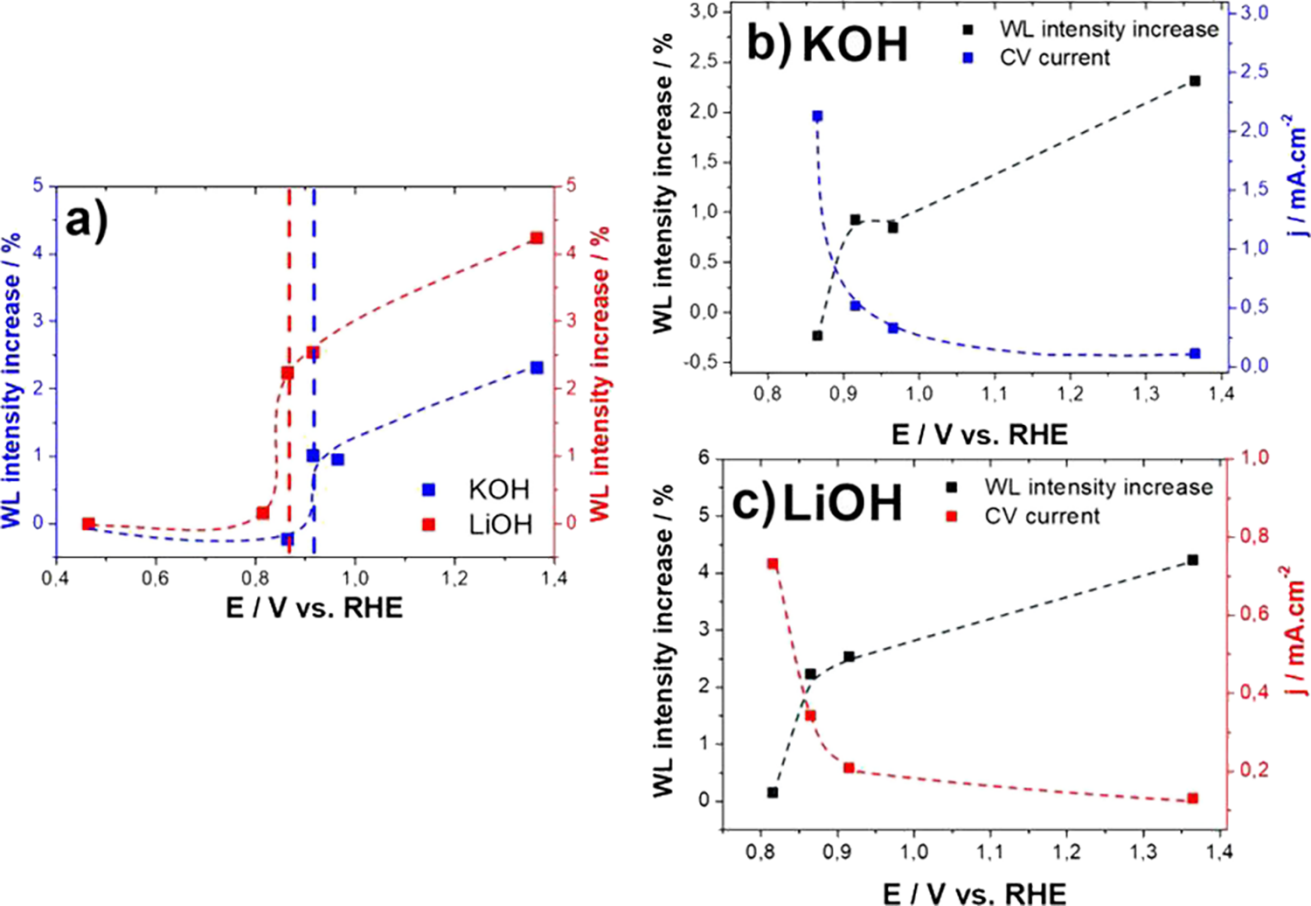
Dependence of the WL intensity on the applied potential in 0.5 M KOH (blue) and 0.5 M LiOH (red) both with 0.1 M GlOH (a). The WL intensity is taken as the peak value in the normalized X-ray absorption spectra presented in Figure S11. The WL increase is calculated as the intensity increase with respect to the value at 0.465 V_RHE_. The experimental points are followed by a B-spline. Red and blue dashed lines highlight the onset of the increase in WL intensity for Li and K, respectively. Comparison between the potential dependence of the WL intensity increase (black line) and of the current density (blue line in (b) and red line in (c)) in 0.5 M KOH + 0.1 M GlOH (b) and in 0.5 M LiOH + 0.1 M GlOH (c).

Initially, we analyzed the WL intensity variation using only the supporting electrolyte. We observed the expected WL increase when going from 0.08 to 0,98 V_RHE_ (Figure S10). Then, we collected XAS spectra around the potential window where the current for glycerol oxidation drops in the presence of glycerol. The WL intensity is observed to clearly increase at 0.865 V_RHE_ in 0.5 M KOH + 0.1 M GlOH, while in 0.5 M LiOH + 0.1 M GlOH such increase occurs already at 0.815 V_RHE_ (Figure 4a), that is, 50 mV more negative than for KOH. The normalized XAS spectra for all measured samples are shown in Figure S11. As clearly shown in Figure 4b,c, where the WL intensity change is compared with the current in the CVs during the EOG, the current drop and the increase int the WL intensity are concomitant. (The CVs from which the current values were extracted are shown in Figure S12).

This suggests that the formation of the inactive Pt oxide layer in the presence of GlOH occurs more negative when using Li^+^ than with K^+^, which is in contradiction with the conclusions drawn solely from the corresponding blank CVs (Figure S2) but is consistent with the ATR-SEIRAS data presented above.

## Discussion

We have shown through combining cyclic voltammetry with *in situ* ATR-SEIRAS and XAS that the relatively sudden current drop after the voltammetric peak during the EOG is due to the formation of an inactive Pt oxide layer, in agreement with early proposals regarding the deactivation of Pt electrodes during the oxidation of small organic molecules due to the formation of a surface oxide layer.^44,45^ We attribute the different activities observed with different electrolyte cations to the effect the cation has on the potential and rate at which that oxide layer forms under otherwise identical conditions. Although this can be explained in terms of noncovalent interactions between the electrolyte cation and specifically adsorbed species on the electrode surface, we wish to highlight that our explanation is different from the merely steric effect proposed 15 years ago by Strmcnik et al.,^17^ according to which clusters of hydrated cations interacting noncovalently with adsorbed OH block the access of the reactant to the Pt surface.

The presence of GlOH and other alcohols in the electrolyte provokes a positive shift in the formation of the oxide layer on Au,^2^ Pt,^46^ and PtNi nanoparticles.^47^ This has been interpreted as being due to the competition between the oxidation of GlOH (to CO_ad_ and other products), the oxidation of CO_ad_ and the formation of a surface oxide, for the same reaction intermediate, namely, OH_ad_.^2,46^ The coincidence of the complete oxidation of CO_ad_ with the current drop suggests that, once CO_ad_ has been completely removed from the surface, oxidation of GlOH to products other than CO_ad_ cannot outcompete the formation of the Pt oxide layer for OH_ad_. In other words, the different activities observed for the EOG in LiOH, NaOH and KOH must be related to the effect that the cation has on the balance between the rate at which OH_ad_ is formed and the rate at which it is removed, either by the oxidation of GlOH and CO_ad_ or the by formation of the surface oxide layer.

The effect of noncovalent interactions between the electrolyte cation and specifically adsorbed species on proton-coupled electron transfer (PCET) reactions involving those species (OH_ad_ formation, GlOH and CO_ad_ oxidation, and the oxide layer formation are all PCETs) has been quantitatively studied and modeled by one of us.^48−50^ According to that body of work, the effect of the electrolyte cation on reactions involving at least one PCET to a specifically adsorbed species is 2-fold: (i) Above a cation-dependent threshold concentration, the electrolyte cation will provoke a concentration-dependent negative shift of the potential at which the PCET occurs, due to the energy required to remove the cation adlayer before the proton can access the plane where the hydrogenation occurs.^48,49^ This purely thermodynamic effect, by which the apparent adsorption energy of the dehydrogenated form of the specifically adsorbed species (*e.g.*, OH_ad_ or the surface oxide layer) increases, was the only one taken into account by Strmcnik et al.;^17^ (ii) below that concentration threshold, the potential at which a PCET occurs will shift positively compared to the cation-free solution due to the effect that the cation’s hydrodynamic radius will have on the location of the outer Helmholtz plane (OHP) and, through it, on the actual electrostatic potential at the plane where the proton transfer occurs (which will roughly coincide with the inner Helmholtz plane, IHP). This will also lead to a super-Nernstian shift of the PCET potential with the solution pH. The smaller the cation’s hydrodynamic radius, the smaller the separation between the IHP and OHP, the larger the positive shift of the PCET potential, and the more super-Nernstian the shift of this potential with pH. This model explains the clear positive shift of the CO-stripping peak on Pt as compared with the cation-free electrolyte when adding as little as 10^–4^ M Li^+^, Na^+^ or K^+^ to a 0.1 M H_2_SO_4_ electrolyte, and that this shift is larger with Na^+^ and K^+^ (smaller hydrodynamic radius) than with Li^+^.^50^ This effect is reproduced in Figure 3, where CO_ad_ is completely oxidized earlier in the positive-going scan in LiOH than in NaOH and KOH, which is then followed in all three cases by the current drop caused by the formation of the Pt oxide layer. Our *in situ* XAS results confirm that the oxide layer forms at less positive potentials with Li^+^ present in the solution than with K^+^.

ATR-SEIRAS also reveals that the anodic peak in the negative-going scan of the CV corresponds to the potential at which the rate of formation of the CO adlayer is maximum across all systems (Figure 3(d–f)), indicating that oxidation of GlOH to CO_ad_ is the main contributor to the current density in this potential region. CO_ad_ is formed at a more positive potential in the negative-going scan in KOH (0.9 V_RHE_), followed by NaOH (0.85 V_RHE_) and LiOH (0.8 V_RHE_). This is consistent with the observation in the positive-going scan that CO_ad_ oxidation is completed at more negative potentials in LiOH, followed by NaOH and KOH, and corroborates our proposed effect of the electrolyte cation stabilizing surface oxygenated species. As explained in the preceding paragraphs, we attribute this apparent stabilization to the effect of the cation on PCET reactions. The latter also offers an explanation to the repeatedly reported stabilization and enhanced adsorption of OH_ad_ by the electrolyte cation (Li^+^ > Na^+^ > K^+^).^31,33^

The cation-dependent location of the OHP, partly responsible for the cation-dependent effect on PCETs described above, is confirmed by the effect of the electrolyte cation on the potential dependence of the CO_L_ frequency in the negative-going scan at *E* < 0.5 V_RHE_. (Although the frequency of the CO_B+M_ band also depends on *θ*_CO_ and the applied potential, identifying its peak frequency is difficult and not free from ambiguities because it contains at least two contributions and is rather wide, so we prefer to limit ourselves to analyzing the evolution during the CV of the frequency of CO_L_ band.) At 0.5 V_RHE_, the highest *θ*_CO_ has been reached in all cases, and from there on, the changes in the CO_L_ frequency shown in Figure S13 (empty symbols) are due exclusively to the Stark effect. We found that the Stark tunning rate is 60, 86, and 119 cm^–1^ V^–1^ (Figure S14) for LiOH, NaOH, and KOH, respectively. This dependence stems from the distinct hydrodynamic radii of the cations, which decreases as we go down the alkaline-metals group, thereby decreasing the distance between the electrode surface and the outer Helmholtz plane (OHP). For the same applied potential, and therefore the same potential difference between the electrode surface and the bulk of the electrolyte, changing the location of the OHP must lead to changing the interfacial electric field, as depicted in Figure S15 and demonstrated recently.^40^ We also attribute the cation dependence of the CO_B+M_ to CO_L_ ratio at the negative potential limit to the cation-dependent intensity of the electric field across the electrical double layer. As discussed in previous work^50^ and summarized above, locating the OHP closer to the IHP will result in a larger positive shift of the PCET potential, *i.e.*, OH_ad_ will form at more positive potentials in the presence of K^+^ than Li^+^.

**Figure 5:**
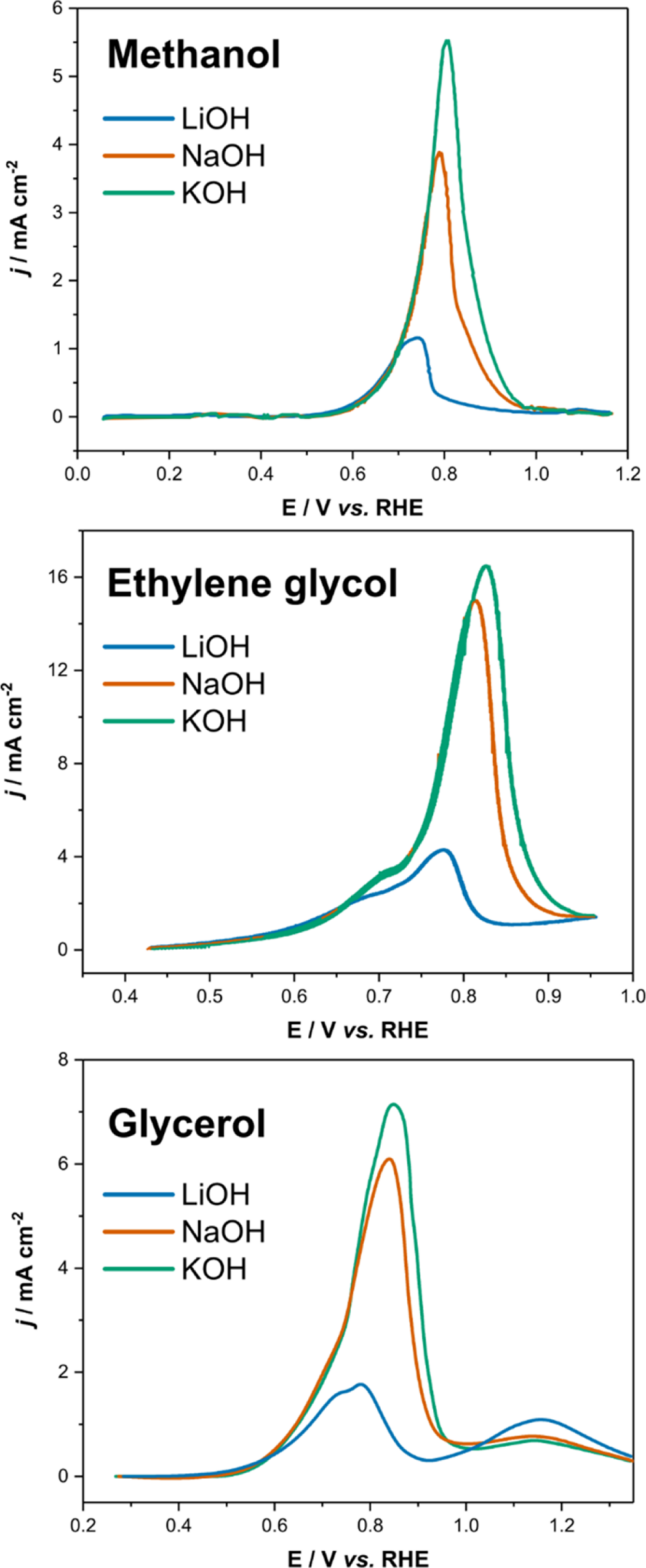
(Top) CV of the electro-oxidation of methanol on Pt(111). (top) Adapted with permission from ref (17). Copyright 2009 Springer Nature. (mid) CV of the electro-oxidation of ethylene glycol on Pt_poly_. (mid) Adapted with permission from ref (19). Copyright 2011 Royal Society of Chemistry. (bottom) CV of the EOG on Pt_poly_. (bottom) Adapted with permission from ref (16). Copyright 2021 Elsevier.

As evidence of the general applicability of the proposed explanation for the effect of cations on the electrooxidation of alcohols and polyols, Figure 5 shows the CVs of the electro-oxidation of methanol on Pt(111),^17^ ethylene glycol on Pt_poly_^19^ and glycerol on Pt_poly_.^16^ In all cases, the effect of changing the electrolyte cation is the same, and differences only emerge when the electrode is deactivated due to the Pt oxide layer formation. Before such deactivation, the CVs are essentially indistinguishable. Additional evidence was obtained by some of us in a previous study, where we showed that the cation does not have any influence on the product distribution of the EOG on Pt in alkaline media.^16^

We have also explored if the deactivation is generated by the adsorption of products/intermediates other than CO_ad_. For instance, carbonate, which is the product of the complete oxidation of GlOH in an alkaline medium that could block active surface sites. However, experiments performed adding Na_2_CO_3_ solution to achieve 1 mM Na_2_CO_3_ in a 0.1 M GlOH + 0.5 M NaOH system are undistinguishable to those in the absence of CO_3_^2–^ (Figure S16).

## Conclusions

In this work, we have combined cyclic voltammograms in glycerol-containing alkaline solutions with ATR-SEIRAS and XAS to explore the effect of the electrolyte cation on the activity of Pt toward the EOG. We conclude that the cation-induced differences in platinum’s electrocatalytic activity for this reaction are due to the effect of the cation on the potential at which the Pt surface deactivates due to the formation of an inactive surface oxide layer, which follows the trend Li^+^ > Na^+^ > K^+^. The formation and electrooxidation of CO_ad_ also follow this trend.

For the inactive Pt oxide layer to be formed, it is necessary to remove from the electrode surface those reactants and/or intermediates that compete with oxide formation for its precursor (OH_ad_). Here we tracked CO_ad_. As the oxidative removal of CO_ad_ follows the trend Li^+^ being less positive than Na^+^, and less positive than K^+^, then the electrode deactivation follows the same trend. We have explained this trend as a combination of the strength of the noncovalent interaction of the cation with OH_ad_ and the effect of the size of the hydrated cation on the potential at the plane where the proton transfer to OH_ad_ occurs, which leads to a stabilization of both OH_ad_ and the surface oxide layer that follows the trend Li^+^ > Na^+^ > K^+^. We have also shown that the same trend is observed in the electro-oxidation of alcohols and polyols in general, where the same explanation applies.

In conclusion, the cation-induced differences in the activity of Pt toward the EOG as well as other alcohols and polyols arise due to the differences in the potential where the deactivation starts rather than to the previously proposed blockage of the access of the reactant to the surface by cation clusters.

## Supporting Information Available

Blank voltammetry of the Pt film with the different cations; ATR-SERIAS series spectra for LiOH and NaOH; Schematic of CO_ad_ on Pt; spectra showing the potential where all CO_ad_ is oxidized; unfiltered intensity of CO_ad_ and H_2_O; XAS spectra; CV using Pt nanoparticles; evolution of CO_L_ peak frequency; stark effect; schematic of the electrochemical interface and EOG in the presence of carbonate (PDF)
