# Supplementary material for: Alkaline-Metal Cations Affect Pt Deactivation for the Electrooxidation of Small Organic Molecules by Affecting the Formation of Inactive Pt Oxide
Source: J Am Chem Soc. 2024 Sep 26;146(40):27745–54. doi: 10.1021/jacs.4c09590 (PMC11467990; doi:10.1021/jacs.4c09590)
Supplement: Supplementary file 1 — ja4c09590_si_001.pdf [file ja4c09590_si_001.pdf]

Supporting Information

Alkaline-metal cations affect Pt deactivation for the electrooxidation of small organic molecules by affecting the formation of inactive Pt oxide

Victor Y. Yukuhiro,[a,b] Rafael A. Vicente,[a,b] Pablo S. Fernández,[a,b]* Angel Cuesta[c,d]*

a. Chemistry Institute, Universidade Estadual de Campinas (UNICAMP), 13083-970, Campinas, São Paulo, Brazil.
b. Center for Innovation on New Energies (CINE), Universidade Estadual de Campinas, 13083-841, Campinas, São Paulo, Brazil.
c. School of Natural and Computing Sciences, University of Aberdeen, Aberdeen AB24 3UE, Scotland, UK
d. Centre for Energy Transition, University of Aberdeen, King's College, AB24 3FX Aberdeen, Scotland, UK

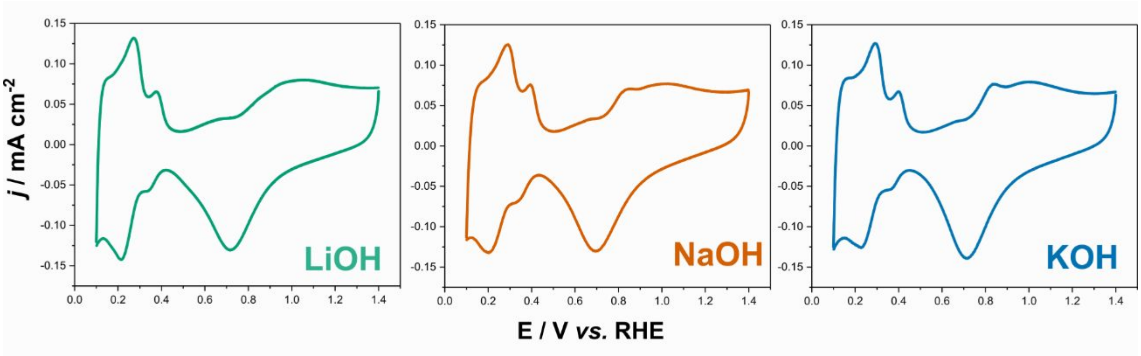

Figure S1. Typical cyclic voltammograms at 100 mV s^{-1} of Pt films deposited on Si in 0.5 M LiOH (left), NaOH (center), and KOH (right).

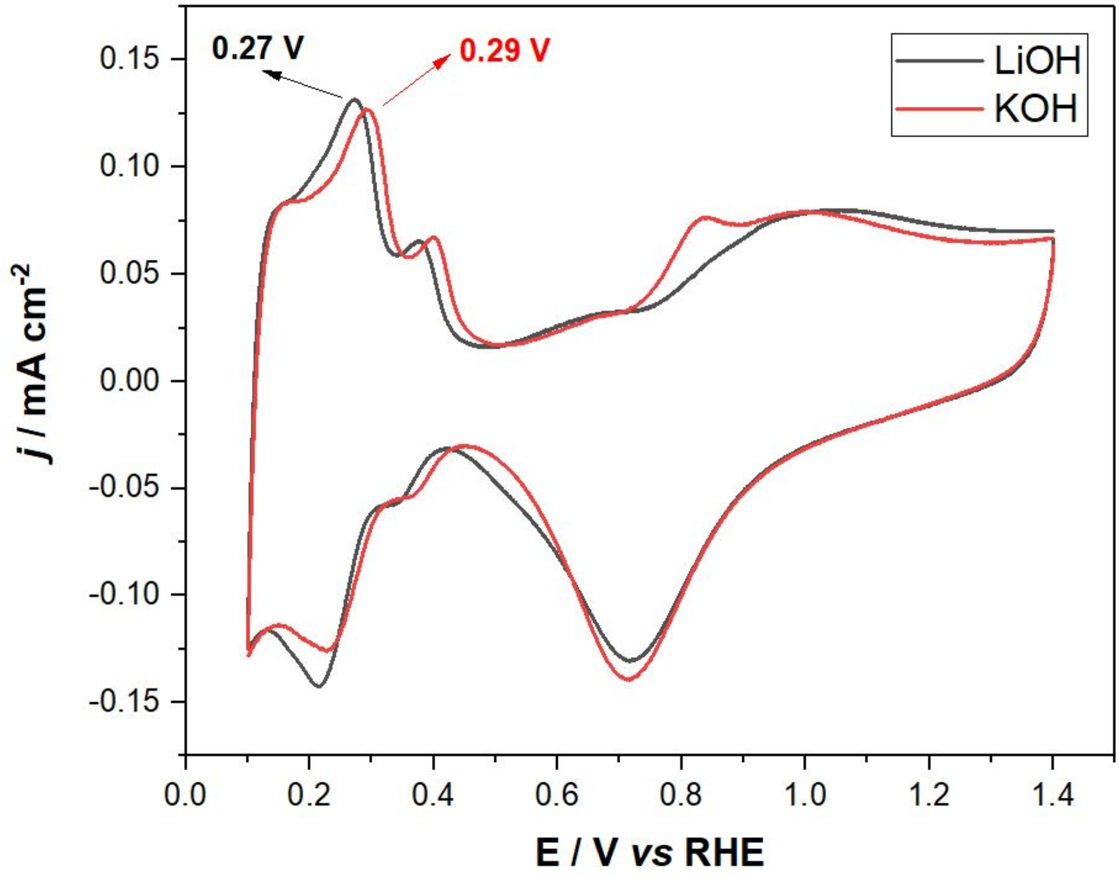

Figure S2. Pt Cyclic Voltammetry at 100 mV s^{-1} in 0.5 M LiOH and KOH.

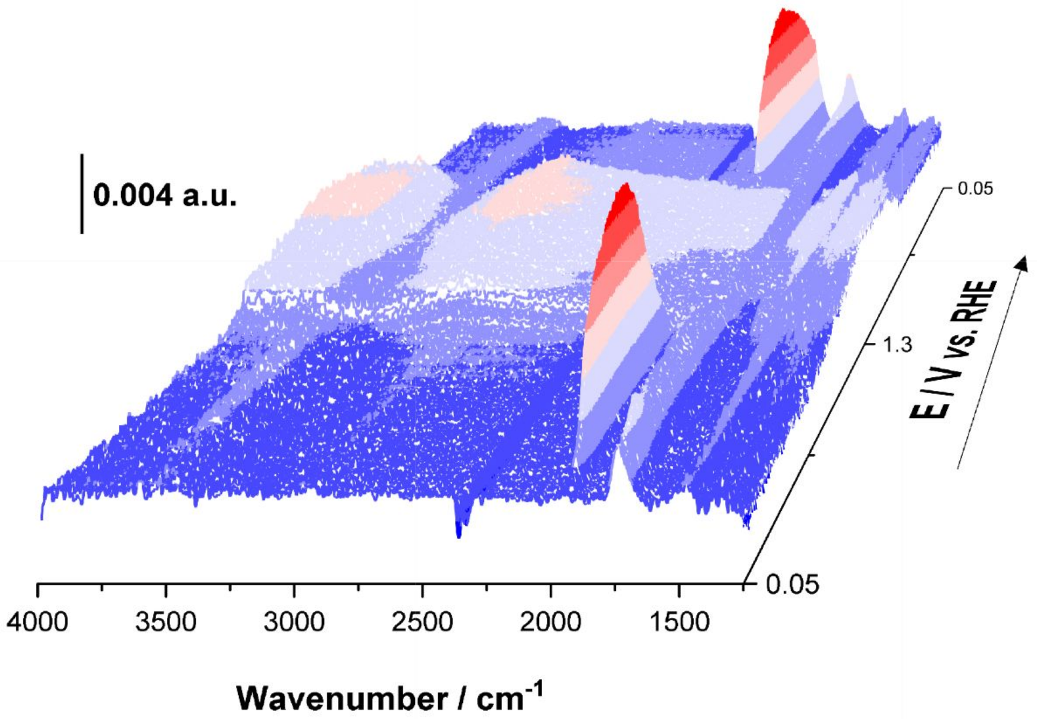

Figure S3. a) Typical time-resolved ATR-SEIRAS spectral series obtained in 0.5 M LiOH + 0.1 M GlOH. Each spectrum is composed of 4 interferograms with a spectral resolution of 8 cm^{-1}. The background is composed of 64 interferograms with a spectral resolution of 8 cm^{-1} and was acquired at 0.05 V_{RHE} in the absence of GlOH.

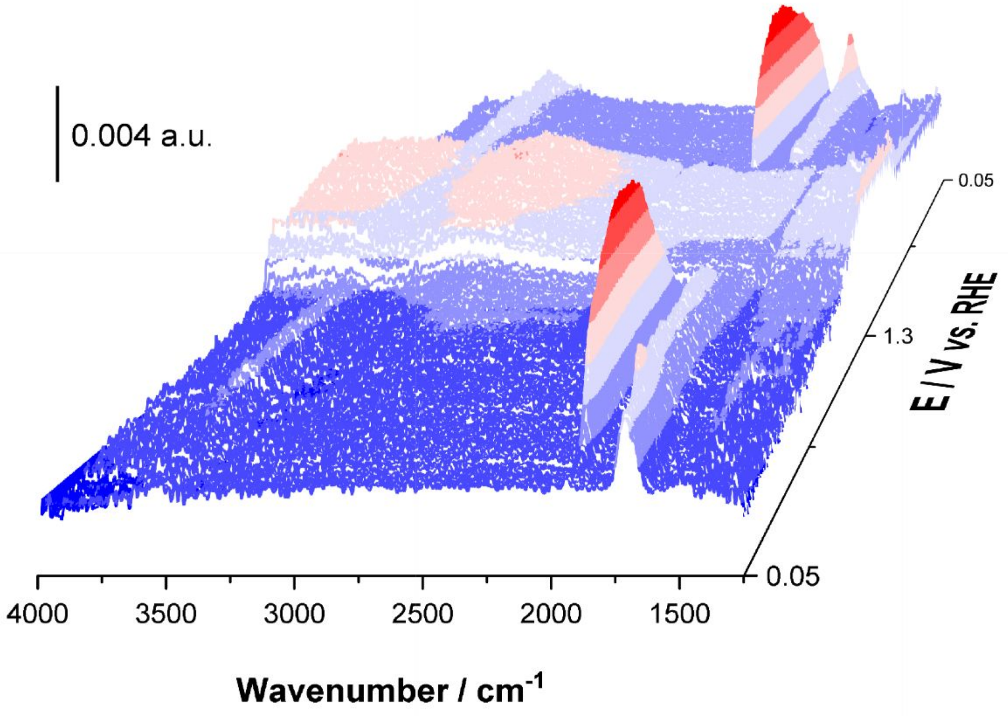

Figure S4. a) Typical time-resolved ATR-SEIRAS spectral series obtained in 0.5 M NaOH + 0.1 M GlOH. Each spectrum is composed of 4 interferograms with a spectral resolution of 8 cm^{-1}. The background is composed of 64 interferograms with a spectral resolution of 8 cm^{-1} and was acquired at 0.05 V_{RHE} in the absence of GlOH.

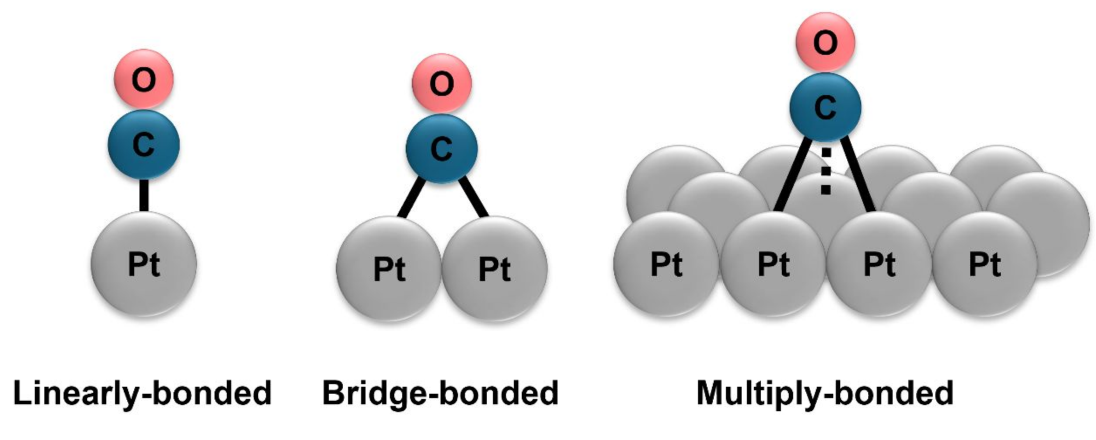

Figure S5. Schematic of the different CO_{ad} coordination on Pt surface.

Fig. S6 shows the potential window where can be observed the CO_L band (~2000 cm^{-1}) and CO_{B+M} band (~1800 cm^{-1}). To obtain the red (CO_L) and blue (CO_{B+M}) dashed lines, we selected the potential at which there is no band in the spectrum e.g., for LiOH (Fig.S6 a) there is no CO_L band at 0.92 V_{RHE}, while at 0.915 V_{RHE} the band was there.

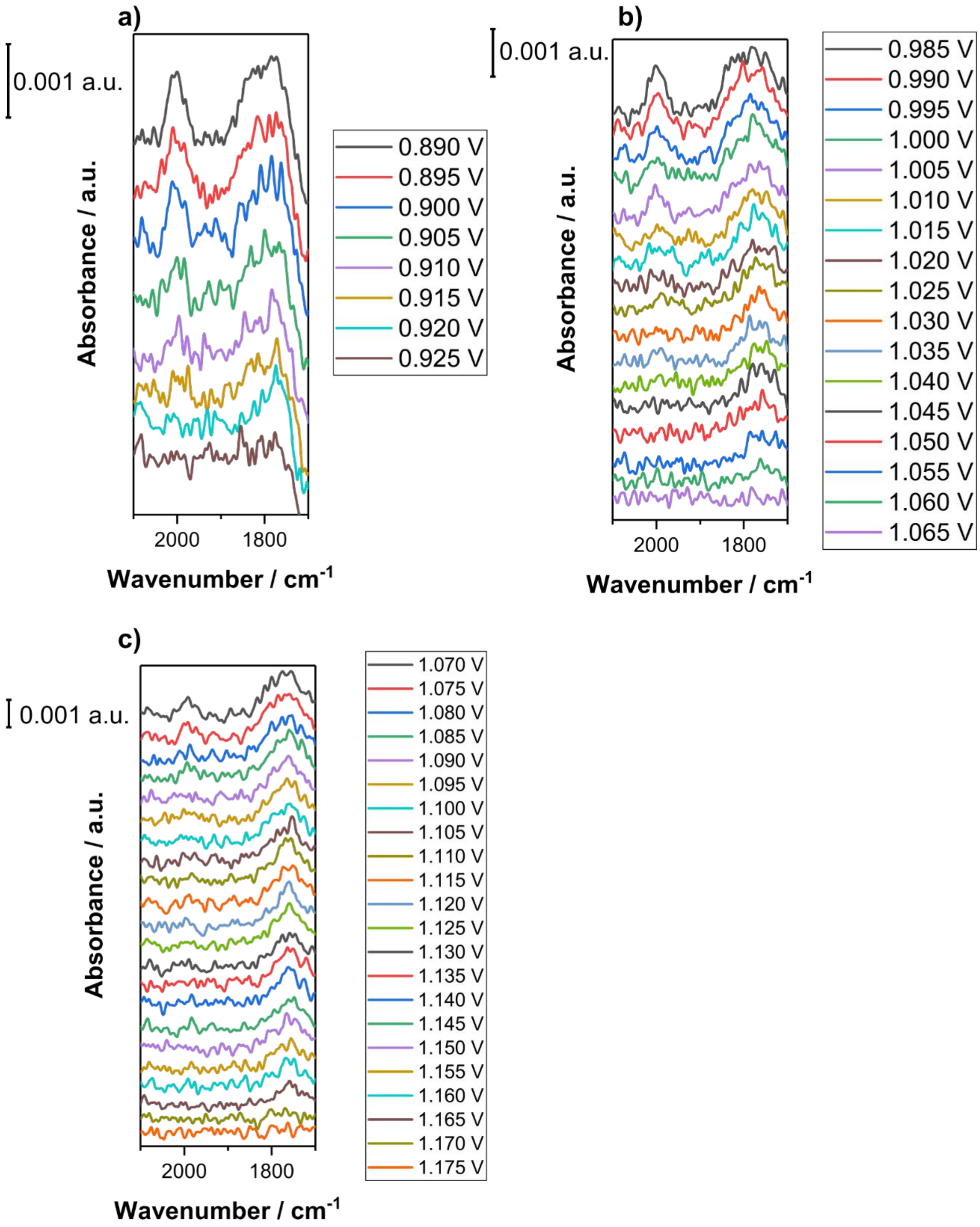

Figure S6. ATR-SEIRAS spectra at a potential range where all CO is oxidized from the EOG series. (a) 0.5 M LiOH + 0.1 M GlOH, (b) 0.5 M NaOH + 0.1 M GlOH, (c) 0.5 M KOH + 0.1 M GlOH. The spectra are composed of 4 interferograms per spectrum with a spectral resolution of 8 cm^{-1}. Backgrounds were acquired at 0.05 V_{RHE} in the absence of GlOH.

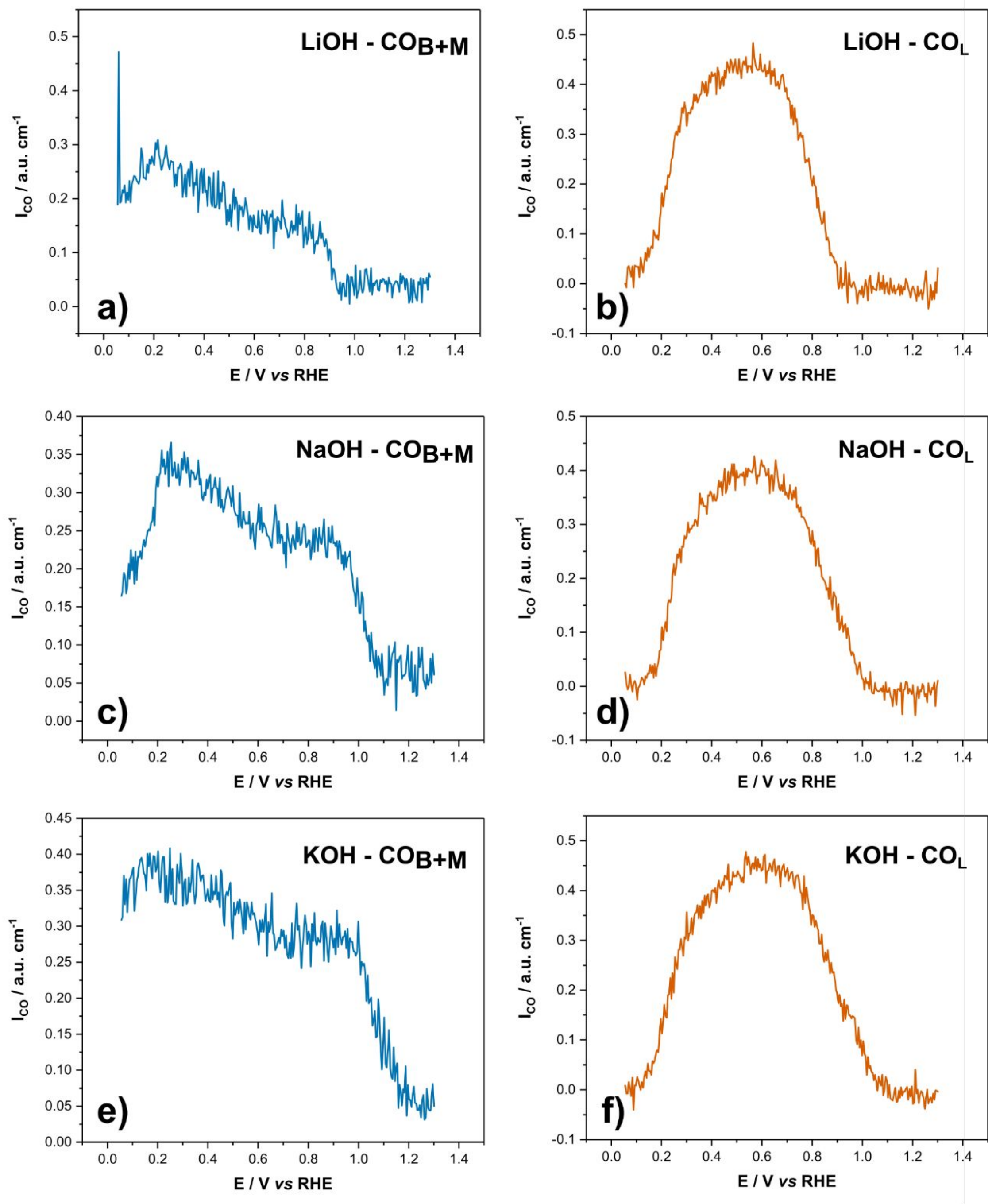

Figure S7. The unfiltered intensity of CO_{B+M} (a, c, and e (blue)) and CO_L (b, d, and f (orange)) from the positive-going scan for all systems.

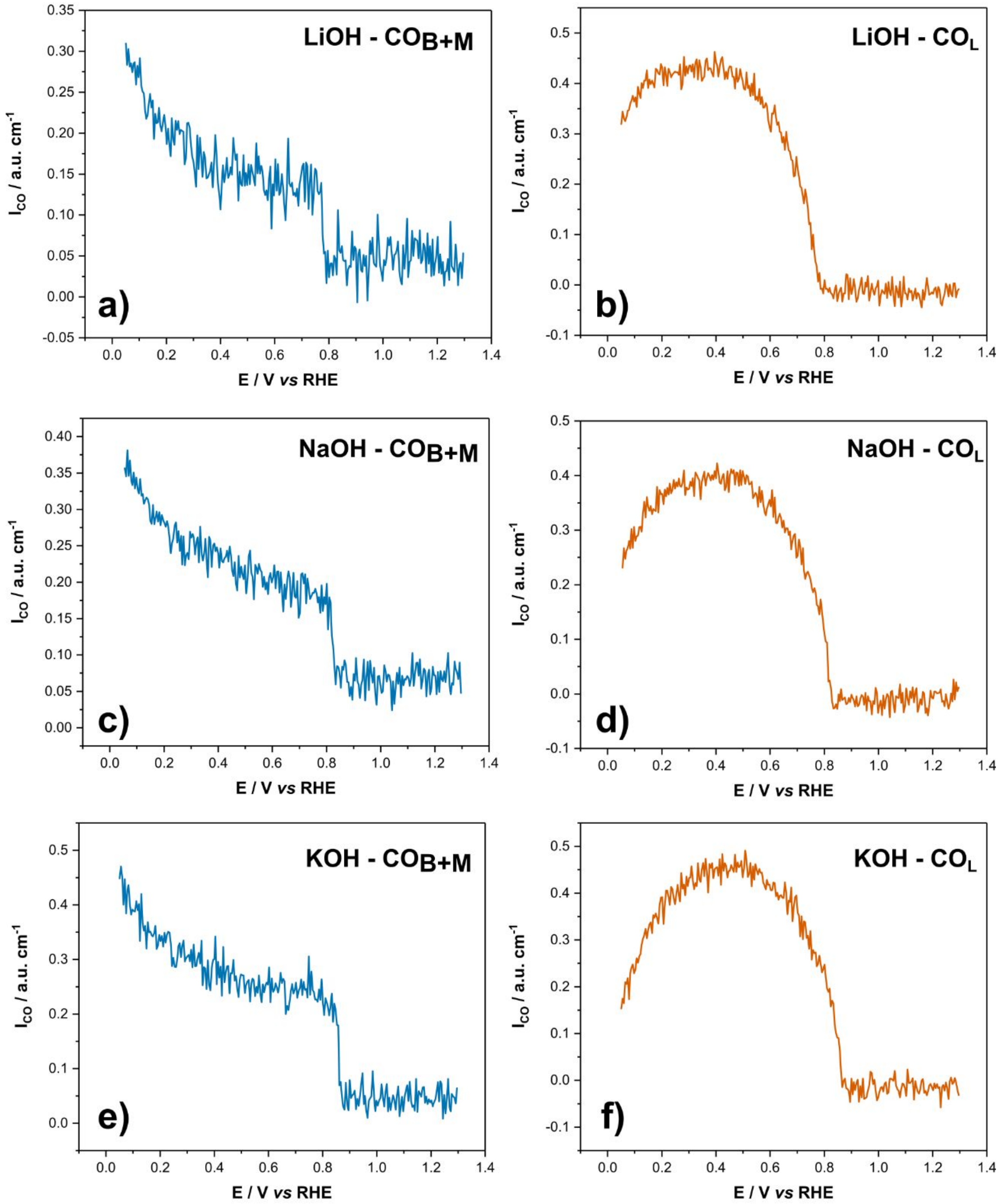

Figure S8. The unfiltered intensity of CO_{B+M} (a, c, and e (blue)) and CO_L (b, d, and f (orange)) from the negative-going scan for all systems.

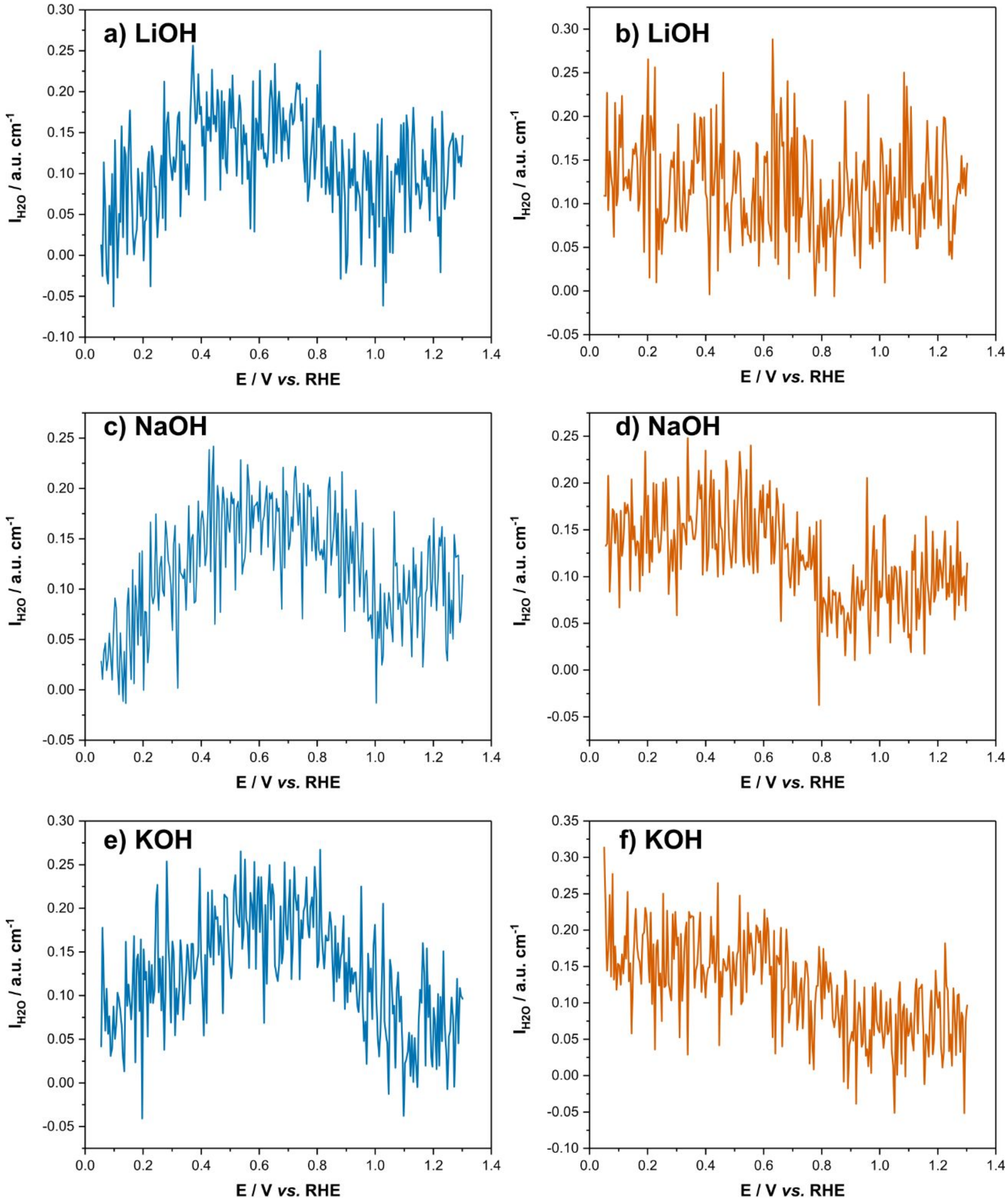

Figure S9. The unfiltered intensity of the H_2O band for the positive- (a, c, and e (blue)) and negative- (b, d, and f (orange)) going scan for all systems.

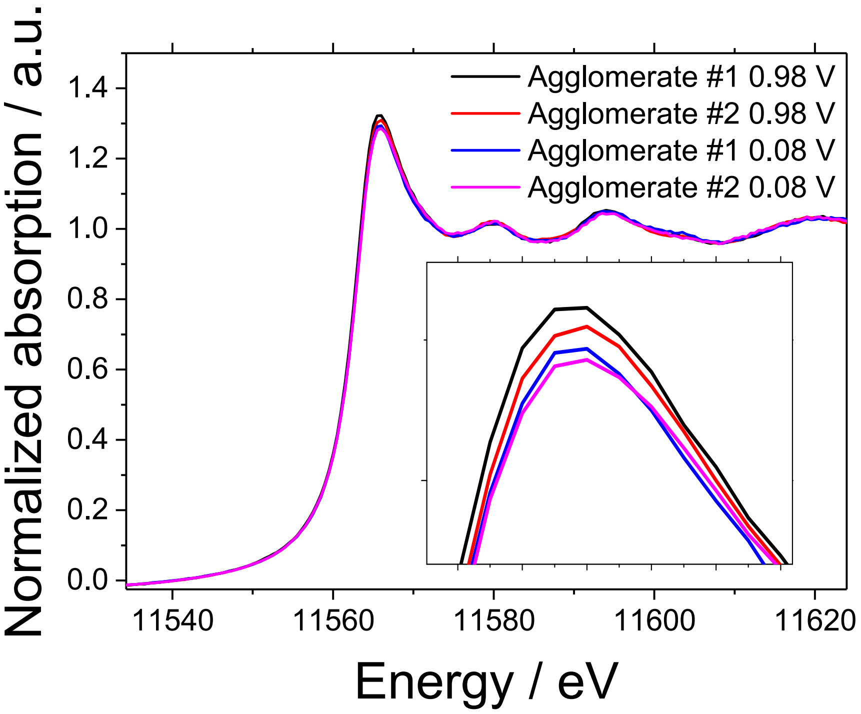

Figure S10. Normalized X-ray absorption spectra for in the absence of glycerol for two different Pt nanoparticle agglomerates showing the increase in white line intensity. The electrolyte is NaOH 0.5 M.

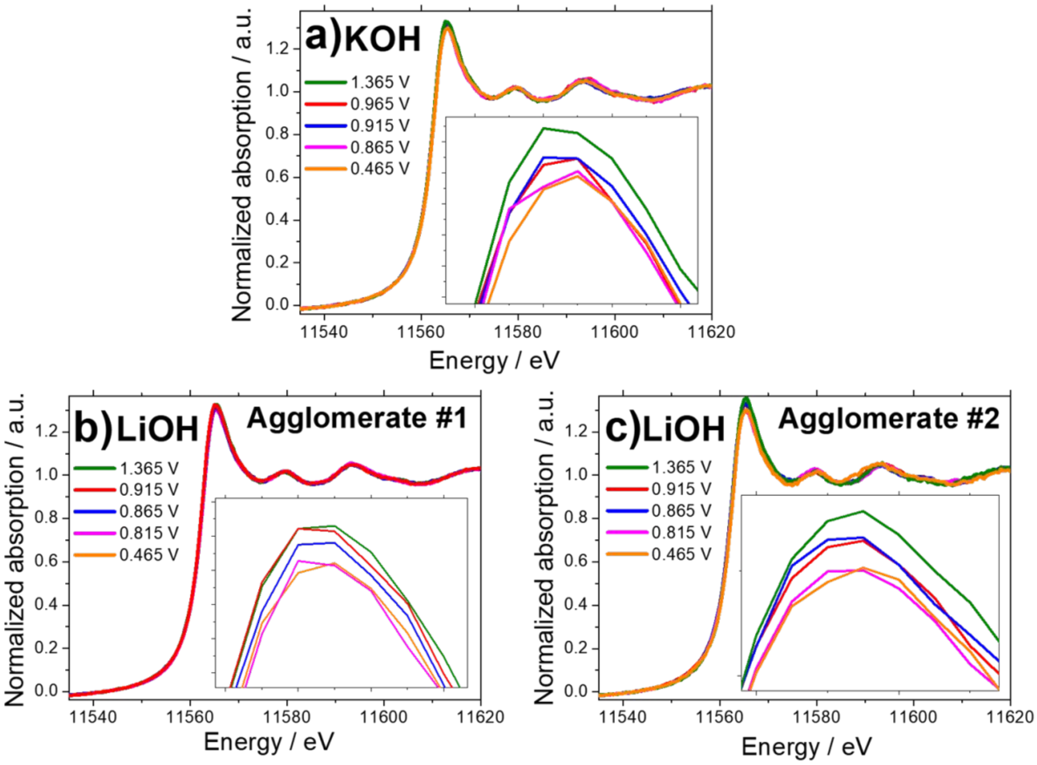

Figure S11. Normalized X-ray absorption spectra for all analysed samples. (a) Using KOH as the electrolyte. (b) Using LiOH as the electrolyte for agglomerate number 1. This is just additional data

to show the data reproducibility. (c) Using LiOH as the electrolyte for agglomerate number 2. This is the data we used in the main article. The inset shows a zoomed in region around the peak of the white line. The white line intensity was extracted at the value where the derivative equals 0 for all samples.

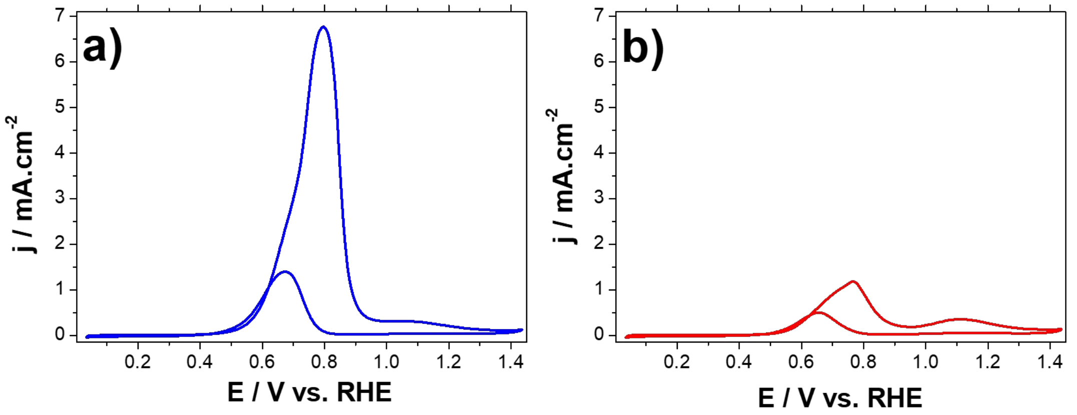

Figure S12. CV profiles used for the comparison with the WL intensity change. These CVs were obtained in the same conditions as the *in situ* experiments, that is, using Pt nanoparticles over a glassy carbon electrode. Scan rate = 10 mV.s^{-1}. The current was normalized using hydrogen underpotential deposition. (a) CV for KOH. (b) CV for LiOH.

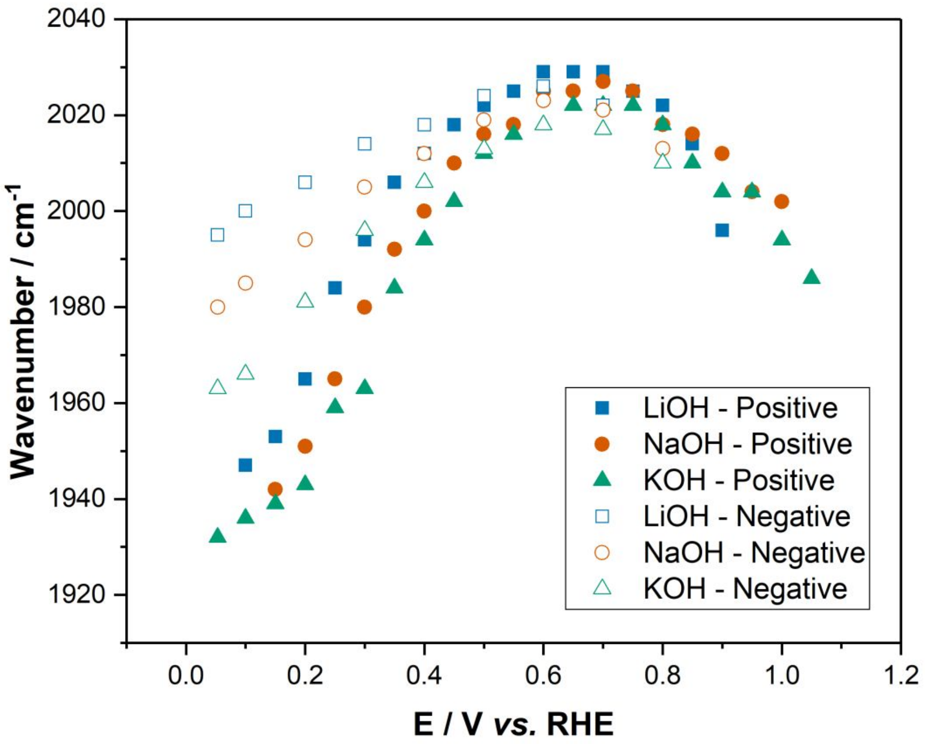

Figure S13. Evolution of the peak frequency of the CO_L during the positive-going (full symbols) and negative-going (empty symbols) scans of a cyclic voltammogram. Black squares, orange circles, and blue triangles correspond to experiments in LiOH, NaOH, and KOH, respectively.

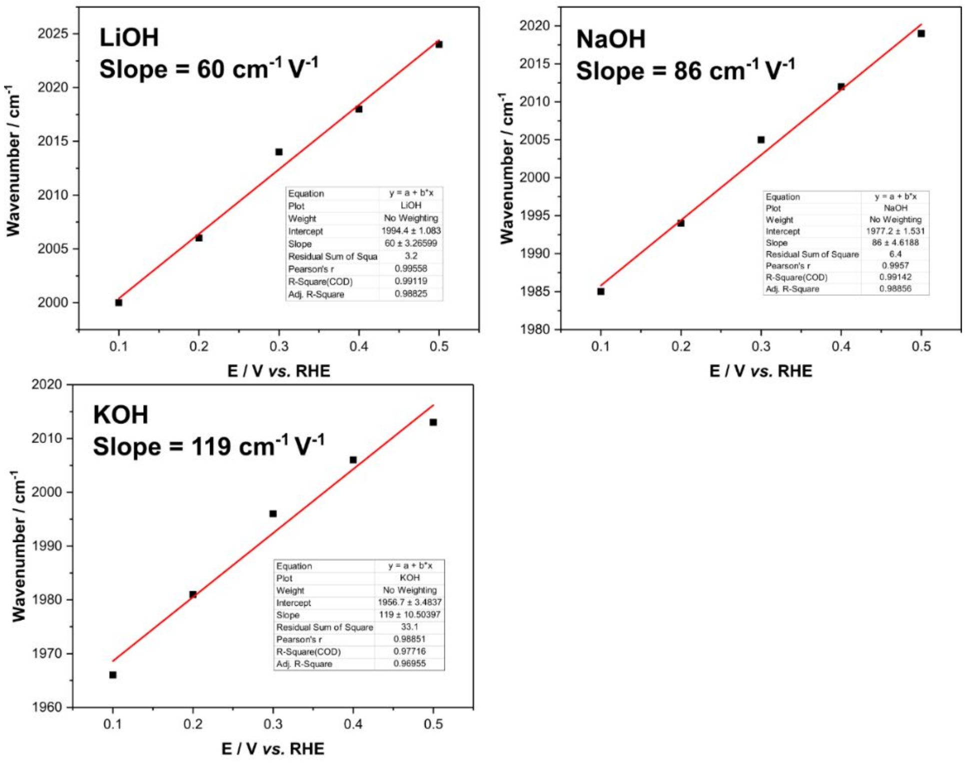

Figure S14. Stark tunning rate for the negative-going scan of CO_L in LiOH, NaOH, and KOH.

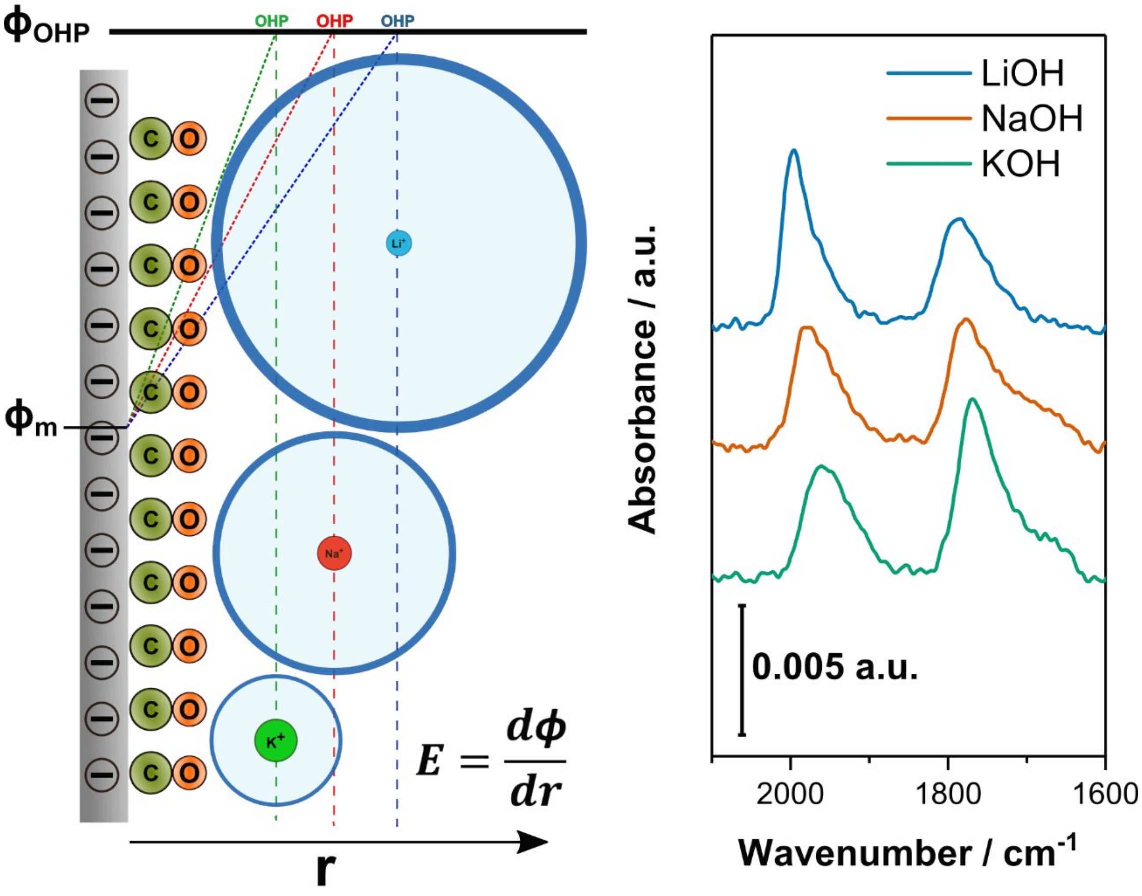

Figure S15. Left: Schematic depiction of the electrochemical interface. The three cations employed in this work are shown as green (K^+), red (Na^+), and blue (Li^+) balls. There are light blue circles around them represent their solvation shells, emphasizing the differences in their hydrodynamic radii. The electrode is a negatively charged, CO_{ad}-covered Pt surface with Galvani potential (ϕ_m). Its charge density is neutralized by solvated cations at the OHP, the location of which is determined by the cation's hydrodynamic radius. The Galvani potential at the OHP is that in the bulk of the electrolyte (ϕ_s) and is made $\phi_s = 0$ for the sake of simplicity. Right: Spectra at the CO region for the three cations at 0.05 V_{RHE} for the negative-going scan.

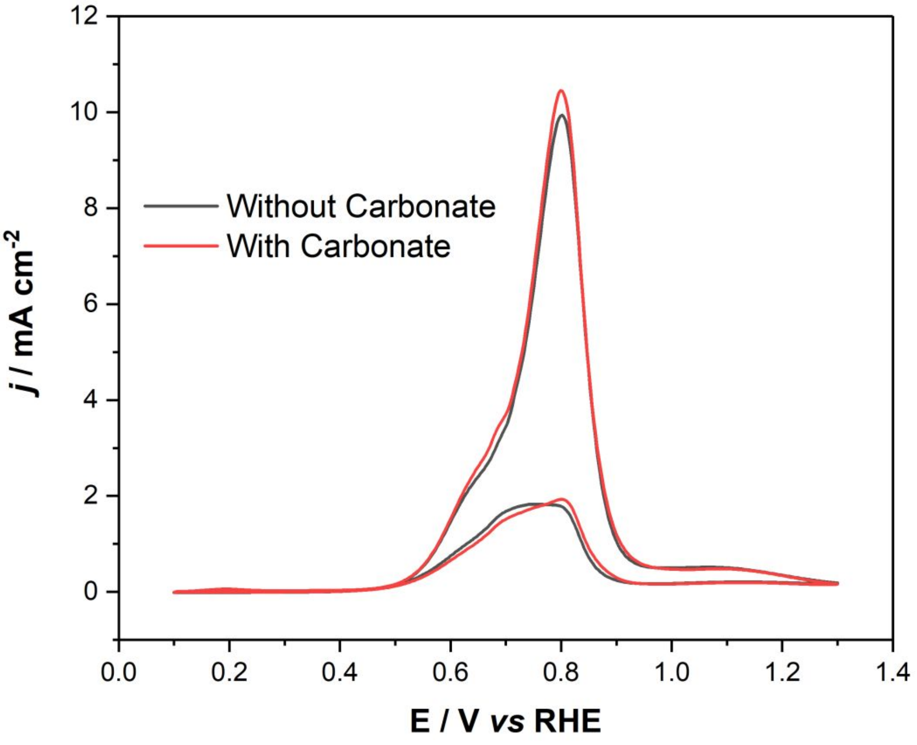

Figure S16. CV of the electrooxidation of glycerol in 0.1 M GlOH + 0.5 M NaOH in presence (red) and absence (black) of carbonate at 10 mV s^{-1}. Reagents: GlOH (≥ 99.5%, Sigma-Aldrich), NaOH (pellets, semiconductor grade, 99.99% trace metal basis, Sigma-Aldrich) and sodium carbonate (P.A., Synth).
